# Book Reviews

**Published:** 1799-11

**Authors:** 


### CRITICAL RETROSPECT OF MEDICAL AND PHYSICAL LITERATURE.

### [FOREIGN AND DOMESTIC.]

*A Third Dissertation on Fever, Part II.; containing an Inquiry into the Effects of the Remedies which have been employed with a View to carry off regular continued Fever, without leaving it to pursue its ordinary Course.* By G. FORDYCE, M.D. F.R.S. &c. 8vo. pp. 200. Johnson, 1799.

THE author of this Dissertation has long been known in the medical world, both as a writer and teacher. The volume before us does not appear, in any degree, to derogate from his well-earned fame.

The means of cutting short the progress of Fever, which Dr. F. examines, are, venæsection, cathartics, antimony, and its preparations, which he details, together with his opinion respecting their modus operandi.

He compares ipecacuanha, and combines it with emetic tartar; cold and warm water, internally and externally; on which last he places little reliance. The next means Dr. F. examines, are epispastics, stimulant sudorifics, antispasmodics, antiputrescents, astringents, and decoct. cinchonæ cum infus. rosæ. On all these remedies, their mode of exhibition and action, he makes such observations as will well reward the perusal.

---

*Essay on the Causes, early Signs, and Prevention of Pulmonary Consumption: For the use of Parents and Preceptors.* By THOMAS BEDDOES, M. D. Second edition, much enlarged, 8vo. pp. 340. Price 6s. Longman and Rees. 1799.

Our readers will conclude, from the title of this work, and the persons for whose use it is written, that the *cure* of consumption forms but a small part of the author's design. His object is a more important and useful one, viz. that of preventing the formation of the disease. On this subject, we might extract many valuable hints and admonitions respecting climate, particular professions or callings exempt from the disorder, others remarkably liable to it; diet, dress, age, habit of body, damp walls or beds, cautions, domestic rules for the prevention of consumption, &c. &c.; but as we think the work itself should be in the hands of all parents and guardians, we shall conclude with expressing our cordial approbation both of the design and execution.

---

*Lectures on Diet and Regimen, &c.* By Dr. WILLICH.

[ Continued from our last Number, pp. 301.]

After having commented at large on the mischievous tendency of patent or quack medicines, made some cursory observations on fashionable fashionable complaints, and investigated the nature and functions of the skin, the author concludes the introduction with an Essay on the Physical Education of Children. From this last we shall quote the following passages:

"There is no practice more detrimental to the powers and energy of man in the first period of his evolution, than that of immediately sinking the tender infant in a soft feather-bed. In this situation, all the organs become extremely relaxed, and we lay the foundation of a very serious malady — a *sweating skin*; the source of constant colds, tooth-achs, head-achs, catarrhs, and innumerable other complaints. For these, and similar reasons, I would advise parents to lay their children, from the very hour of their birth, on soft and cooling mattresses, under thin blanket covers, or cotton quilts, which do not incommode the body, leave the hands and arms at liberty, and are not liable to excite too great a degree of heat. In the intense cold of winter, an additional blanket may be used, which, however, should be removed when the weather turns milder, and the child grows stronger. But the greatest mischief arises from bolsters or pillows filled with feathers; which must, after a certain time, produce uncleanliness and a disagreeable smell. Such a pillow is calculated to collect and retain mephitic vapours; and, for this obvious reason, it cannot but be unsafe to sleep for a whole twelvemonth with one's head reposed on such a mass of acrid exhalations. This inconvenience may be easily avoided, by furnishing children with cushions filled with horse-hair, or with the softest bran, previously well beaten; the best for this purpose is the bran of oats. The great advantage of these pillows is, that they admit moisture to pass through them, consequently they will always remain dry, and may from time to time be renewed, while they preserve a moderate and regular degree of warmth.

"*Cleanliness*, in domestic life, is one of the cardinal virtues, and an essential requisite to the proper physical eduction of children. Indeed, I cannot help remarking, that this is, perhaps, the *only* province of parental care, in which we *never* can do *too much*. For this end, we ought not to neglect the article of linen, as the frequent change of it is of more consequence than many parents are aware of. A child is much more liable to perspire than an adult; the natural effect of which is, that its linen is more readily soiled, and rendered unfit for wearing. I would therefore advise all parents, who can afford it, to give their children clean dry linen *every day*. An undoubted proof of the utility of this practice is, that instances have occurred of children being cured of the rickets, when, from the first appearance of that complaint, they have been daily furnished with clean linen, well dried, and occasionally smoaked with juniper berries, frankincense, or other perfuming substances, in order to expel the moisture, which is absorbed by linen. But if a clean change cannot be conveniently had every day, the night-shirt, as well as that of the day, ought to be regularly dried and perfumed if necessary.

"Lastly, let the dress of children be light; the head and breast, during the first months, may be covered, though very slightly; but as soon as the hair is sufficiently strong to afford protection, there is scarcely any necessity for hats or caps, unless in rainy or cold seasons. The breast and neck, too, acquire more firmness, and are rendered hardier by keeping them uncovered; as our frequent colds and sore throats, chiefly originate originate from the abfurd habit of wearing bofom friends and ftiffened cravats.

"I fhall conclude thefe obfervations with an hiftorical anecdote from HERODOTUS, which clearly illuftrates the advantage attending the cool regimen of the head. This judicious and learned writer informs us, that, after the battle fought between the *Perfians*, under CAMBYSES, and the *Egyptians*, the flain of both nations were feparated; and upon examining the heads of the Perfians, their fkulls were found to be fo thin and tender, that a fmall ftone would immediately perforate them; while, on the other hand, the heads of the Egyptians were fo firm, that they could fcarcely be fractured by the largeft ftones. The caufe of this remarkable difference, HERODOTUS afcribes to a cuftom the Egyptians had of fhaving their heads from the earlieft infancy, and going uncovered in all ftates of the weather; whereas the Perfians always kept their heads warm, by wearing heavy turbans.—pp. 92—95.

The whole work is divided into *twelve* chapters: in the firft, the author treats "*On the Means of preferving Health and prolonging Life*."—We extract the following paffage from his enumeration of the conditions neceffary to attain a long and healthy life.

"*Fourthly*, A gradual, and not too precipitate culture of the phyfical and mental faculties may be properly confidered as an excellent preliminary ftep towards prolonging life. The age of man bears a certain proportion to the growth of his various powers; and the longer we can protract the different ftages of life, the more extended will be the whole compafs of our exiftence. As it is evidently the defign of nature, that man fhould live longer than moft of the lower animals, he of courfe requires a greater fpace of time to develope the faculties both of mind and body. Animals, which arrive foon at the perfection of their nature and form, live but a fhort time. Man requires upwards of twenty, and, according to fome, twenty-five years, before he attains to full maturity; and if it be a rule of nature, that animals in general live eight times the number of years which is requifite to the attainment of their perfect growth, a ftrong prefumption arifes, that the age of man might be extended to nearly two hundred years. In the works of the illuftrious BACON, and particularly in his "Hiftorical View of Life and Death," are given many ftrong arguments to confirm this affertion. "Surprifing as it may appear to fome, there is a poffibility, at leaft, if not a probability, that the term of human life might be ftill farther extended, if mankind could by any means be perfuaded to return to that primeval ftate of nature, from which hiftory and tradition have furnifhed us with fuch aftonifhing, and almoft incredible inftances of longevity. It is not my intention here to inquire into the degree of credit, which may be due to the accounts of fome extraordinary facts of individual longevity recorded by the facred hiftorian; as the learned vary much in their opinion, relative to the mode of computation, and whether the Solar, the Arabic, or the Lunar year, or a ftill fhorter meafure of time, is alluded to. This, at leaft, feems to be generally admitted, that the antediluvians enjoyed an enviable, uninterrupted ftate of health; that their vegetable aliment, and general mode of living, were extremely fimple and nowife prejudicial; that the conftitution and temperature of the globe itfelf muft have been greatly affected and deteriorated, in confequence of the flood, or other caufes of which we are ignorant; and laftly, that thofe thofe impetuous and inordinate appetites and paffions, which, like flames, may now be faid to confume the powers of life, were then either lefs violent, or exerted their baneful influence at a much later period of life."

[ To be concluded in our next Number. ]

---

*Memoirs of the Medical Society of London*, vol. v. 8vo. 470 pp. *Johnfon* London, 1799.

When we commenced the analyfis of this work, in the fixth number of our Journal for Auguft, pp. 29—35, we were then affured, that it would be publifhed in the courfe of that month. Unforefeen accidents, however, delayed the publication; and in the mean time, a cafe of Cæfarian fection, from the Lying-in-Hofpital at Manchefter, was communicated to the Society, with an engraving reprefenting the bones of the pelvis, which was thought proper to be annexed to the volume as an appendix. As we have already publifhed feveral papers, tending to difcourage and condemn this mode of delivery, we fhould think it but juftice to the Gentlemen concerned in the prefent inftance, to give a particular account of the cafe, and the dimenfions of the pelvis; all which tend to fhew that this fection offered the only chance of faving the life of the mother. Want of room, at prefent, obliges us to poftpone this act of juftice.

---

*Biographia Medica*; *or, Hiftorical and Critical Memoirs of the Lives and Writings of the moft eminent Medical Characters that have exifted from the earlieft Account of Time to the prefent Period, with a Catalogue of their Literary Productions*; by Benj. Hutchinson, 2 vol. 8vo. each 546 pages, 16s. *Johnfon*. London. 1799.

In this work, the lives of the authors introduced are arranged in alphabetical order. The ftyle is neat and unaffected, and biography has always charms fufficient to fecure readers. On this account, as well as for the convenience of reference, we conclude that few Medical Libraries will be without the Biographia Medica.

---

*Verfuch einer Chronologifchen Ueberficht, &c.*—An attempt to exhibit the literary hiftory of medical fcience, in chronological order, with a view to promote and facilitate its ftudy, by Dr. J. G. Knebel, 8vo. xxxiv. and 377 pp. (1 rixd. 8 grofch. or 5s. 6d.) 1799. *Breflau.* Korn, fenior.

Medicine has long felt the want of a work which would furnifh the medical ftudent with fuch information relative to this fcience, as is contained in "Saxii *Onomafticon*" with refpect to the fciences in general. A work of this nature, however, fhould comprife fomewhat more than a mere catalogue, in which the years of birth and death, the principal refidence and public office held by phyficians, or even their chief works, together with the beft editions, are pompoufly recorded. Neither "*Matthiæ confpectus hiftoriæ medicorum chronologicus*," nor Kestner's Lexion of Medical Literary Men, nor Eloy's work on the fame fubject, (both in German) are fufficient for that purpofe; and the practitioner, who has not accefs to great libraries, and cannot afford to purchafe one for his private ufe, is frequently at a lofs, efpecially if he is defirous desirous to make himself acquainted with the literary history of this science.

Dr. KNEBEL has arranged the present work in chronological order, and, in this respect, imitated Saxii Onomasticon. The whole of this chronological view is divided into four distinct periods: The first extends to HIPPOCRATES; the second to the death of GALEN; the third to PARACELSUS; and the fourth to the year 1797. The physicians still living are, however, not comprised in this retrospect, as the author generally begins his sections from the demise of medical writers. To every chronological table he has prefixed a summary recapitulation of the events and changes that have taken place during the period of which he treats; this retrospective account could be only brief and very imperfect. Several medical characters, among whom are some of considerable reputation, have been omitted; while others of little merit, particularly in the first period, have been inserted. Nor are the names of authors printed with uniform correctness, though the editor has not neglected to give an extensive list of *errata* and *addenda*; for instance, instead of LIPPENIUS we find *Lixenius*, and sometimes *Lipenius*; instead of ERASTUS, *Ernst*; for CHRISTOPH. DE HONESTIS, *de Honestiis*, &c.

Notwithstanding these errors and imperfections, we are not acquainted with a later and better historical work, or one which we might with more propriety recommend to the medical tyro. The author is, doubtless, entitled to some praise and encouragement, that he may, in a subsequent edition, improve his labours on this thorny path.

---

*Tableau du regne vegetal selon la methode de Jussieu.*—A View of the Vegetable Kingdom, according to the method of Jussieu; by E. P. VENTENAT, of the National Institut of France; and one of the librarians (conservateurs) at the Pantheon. Four volumes, together 2158 pp. and 24 plates; 8vo, *Paris, an* 7 (1799) price 21 livres, or 24 liv. free through all the departments. Publ. by the author and Drisonnier.

Although the system of the celebrated Jussieu is one of the most ingenious we possess in Botany, as this author has furnished us with excellent illustrations of many undetermined botanical characters, yet a work was still wanted for the elucidation of obscure passages, and the embellishment of others that were heavy and dry. M. VENTENAT, who is already known to the learned by his *Principes de la Botanique*, on which he gave public lectures in the *Lycée Republicain*, as well as by a variety of essays inserted in different periodical works, has here attempted to treat of all branches of botany in a comprehensive systematic work, grounded chiefly upon the principles of Jussieu. He has carefully availed himself of the observations made by cotemporary botanists, while he has also enriched it with his own remarks, for which he found an ample field in the extensive herbals at Paris, as well as in the celebrated botanical garden belonging to the Museum, and that of M. CEL.

The principal subjects contained in the first volume relate to the philosophy of botany. The author gives not only the terminology of plants, in alphabetical order, according to LINNÆUS, JUSSIEU, GAERTNER, and others, but he illustrates the terms with apposite examples, and accompanies the description of plants with physiological and œconomical remarks. The discoveries of GREW, MALPIGHI, DUHAMEL, BONNET, SAUSSURE, DAUBENTON, SENNEBIER, BERTHOLET, &c.

are are faithfully recorded; and that important branch of botany which relates to agriculture, has received many valuable additions from the ufeful refearches of DUHAMEL, TILLET, TESSIER, ROZIER, PARMENTIER, and other bucolic writers.

The fecond and third volumes of this claffical work contain the genera of plants, or the fyftem itfelf, together with an account of fome of the principal fpecies rarely found in Europe. In defcribing the genera, our author has paid particular attention to thofe botanifts who have determined them, as well as to thofe who have adopted and exhibited them in the moft correct and confpicuous manner. He has alfo fubjoined to every plant its fynonymous and vernacular name. On the union of the different fexes of plants, and on the combination of thofe of the fame fex, M. VENTENAT has made feveral very ingenious obfervations; and as the whole of botanical fcience confifts in properly diftinguifhing thefe combinations and diffimilarities, he has developed this delicate fubject in a manner almoft completely fatisfactory.

In the fourth volume, the author firft treats of fome plants which are remarkable by their peculiarities, and therefore appear to form new orders; but he likewife mentions feveral others which have already, by former botanifts, been comprifed in the number of certain families, for inftance, the *Cufcuta*, *Tozzia*, *Globularia*, *Samolus*, &c. yet he does not neglect to ftate the caufes which induced him to feparate fuch plants from the families in which they had been placed by his predeceffors. Next we meet with an appendix, containing the remarks that occurred to the author fince the work has been committed to the prefs; to which is added, an index of the names, genera, fpecies, and fynonymous terms, in the Latin and French languages; together with a complete lift of the authors and their works quoted in thefe volumes. The plates and the explanations annexed are executed in a fuperior ftyle; they are drawn by the celebrated artift, J. H. REDOUTÉ, and engraved by SELLIER; our limits, however, do not permit us to detail their multifarious contents.

The whole is concluded with a methodical table, which greatly facilitates the retrofpective view of the whole fyftem, and enables the reader to find, without trouble, the proper place and deftination for every plant. Indeed, the retrofpect itfelf is equally eafy; as the general table, in every inftance, refers the reader to the following, which are more particularized: and this table, by comprifing all the genera, renders the work peculiarly valuable; in the compofition of which the induftrious author has beftowed the attention requifite to a correct and elegant performance.

---

*Grundrifs der Phyfiologia Pathologica:*—Elements of pathological phyfiology; or the doctrine of the ftructure, compofition, and functions of the human body and its parts, in a preternatural ftate. Second and laft volume, containing the Natural Functions, by E. F. HECKER, M. D. 8vo. 238. pp. *Halle*, Hemmerde, 1799.

The author began this ufeful fmall work with the firft volume, publifhed as late as the year 1791. In the prefent part, he treats, in his ufual perfpicacious manner, of the appetite for food; of maftication and deglutition; of the great ftomachic gland; of the liver, bile, and gallbladder, when he endeavours to prove at large, that the bile is fubject to rancidity; of the fpleen; the integuments of the abdomen, the peritonæum, ritonæum, the mefentery, and the omentum; of the inteftines; of the fyftem of the lymphatics, where the author gives his opinion at fome length, that the whole alimentary function principally depends upon the lymphatics;—of fanguification; of chylifaction; of the fecretions; and, laftly, of the urinary organs—without however touching on the fexual functions. On every occafion, the learned author has illuftrated his propofitions by quotations from the lateft and moft eminent writers; while, under every head, he has added a lift of the moft important medical works relative to the fubjects under confideration.

---

*Schwedifche Annalen*, &c. The Swedifh Annals of Medicine and Natural Hiftory: Edited by K. A. Rudolphi, M. D. &c. &c. Vol. I. No. I. 8vo. 16 fheets, price 20 grofch, or about 3s. *Berlin*, Lange, 1799.

The editor of thefe Annals is entitled to the thanks of the medical world, for having furnifhed us with a periodical work of fuch extenfive and ufeful tendency. The literature of Sweden has but too long remained to us a *terra incognita*, although the learned of that country have always held a diftinguifhed rank in the literary republic, efpecially in the departments of Natural Hiftory and Phyfics.

Dr. Rudolhpi is very favourably fituated for communicating to us from that quarter, whatever is valuable and interefting; he carries on active correfpondence with feveral literary men of that kingdom; and the Univerfity library of Greifswalde, is daily open to his infpection; while a copy of every work printed in Sweden, is regularly tranfmitted to that German College, where he fills the place of Adjunct to the Medical Faculty, and Anatomical Diffector.

The firft number of this work contains a great variety of effays, critical and analytical, as well as accounts and reviews of new medical works. The moft remarkable among the former, are the following:

I. On the progrefs of anatomy in modern times, by Adolph Murray. II. A defcription of a collection of natural curiofities, by A. U. Grill, of Söderfors; accounts of a living *Simia Apella*, a living *Cavia Aguti*, and an oftrich which died of too great obefity. III. On the hiftory of zoological knowledge in Sweden, prior to the time of Linnæus; by Gust. Paykull. IV. A difcourfe on the eftablifhment and improvement of medical fcience in Sweden, by I. G. Acrel. V. Analyfis of the New Tranfactions of the Royal Academy of the Sciences at Stockholm, for 1797. VI. Analytical account of a work, entitled, "The Phyfician and Naturalift, Vol. XII." Among other inftructive extracts from this work, we met with a letter from Tunis, written in 1795, by H. C. Gersonius, whofe account of the plague is the more interefting, as he fuccefsfully employed the *arnica* againft this formidable difeafe. We are anxious to fee his account of the plague, and lues, which he has promifed to tranfmit to the College of Phyficians at Stockholm.

[ To be continued in our next Number. ]

